# Supplementary material for: Biodiversity increases resistance of grasslands against plant invasions under multiple environmental changes
Source: Nat Commun. 2024 May 27;15:4506. doi: 10.1038/s41467-024-48876-z (PMC11130343; doi:10.1038/s41467-024-48876-z)
Supplement: Supplementary file 1 — Supplementary Information [file 41467_2024_48876_MOESM1_ESM.pdf]

Supplementary Information

Biodiversity increases resistance of grasslands against plant invasions under multiple environmental changes

Cai Cheng[1,2], Zekang Liu[2], Wei Song[2], Xue Chen[2], Zhijie Zhang[3], Bo Li[4], Mark van Kleunen[3,5], Jihua Wu[1 *]

[1]State Key Laboratory of Herbage Improvement and Grassland Agro-Ecosystems, College of Ecology, Lanzhou University, Lanzhou 730000, China

[2]Ministry of Education Key Laboratory for Biodiversity Science and Ecological Engineering, National Observations and Research Station of Wetland Ecosystems of the Yangtze Estuary, Institute of Biodiversity Science and Institute of Eco-Chongming, School of Life Sciences, Fudan University, Shanghai 200438, China

[3]Department of Biology, University of Konstanz, Konstanz 78464, Germany

[4]Ministry of Education Key Laboratory for Transboundary Ecosecurity of Southwest China, Yunnan Key Laboratory of Plant Reproductive Adaptation and Evolutionary Ecology and Centre for Invasion Biology, Institute of Biodiversity, School of Ecology and Environmental Science, Yunnan University, Kunming 650504, China

[5]Zhejiang Provincial Key Laboratory of Plant Evolutionary Ecology and Conservation, Taizhou University, Taizhou 318000, China

*** Correspondence**: Jihua Wu, E-mail: wjh@lzu.edu.cn, Tel & Fax: 0086-931-8915360

Supplementary Methods

In addition to the net biodiversity effect (NBE) on invasion resistance, we also quantified biodiversity effects using the Pearson's correlation coefficient between resident species richness and the performance of invaders, which is also widely used for quantifying biodiversity effects in diversity–invasion resistance experiments[1,2]. We inverted the sign of the correlation coefficient because a high performance of invaders indicates low invasion resistance. Then, we normalized the inverted correlation coefficient using Fisher's z-transformation to obtain the effect size of biodiversity effects (BE_{Zr}) on invasion resistance[3]:

$$BE_{Zr} = \frac{1}{2}\ln\left(\frac{1+r}{1-r}\right),$$

Supplementary Equation (1)

where r is the inverted correlation coefficient. The variance of BE_{Zr}, v_{BE}, was calculated as[3]:

$$v_{BE} = \frac{1}{n-3},$$

Supplementary Equation (2)

where n is the sample size. We also calculated resident-productivity BE_{Zr} using Supplementary Equation (1) with the raw correlation coefficient between resident species richness and resident productivity.

Likewise, we calculated the difference in invasion-resistance BE_{Zr} between ambient and manipulated environmental conditions (ΔBE_{Zr}) and its variance ($v_{\Delta BE}$) using the following equations[4]:

$$\Delta BE_{Zr} = (BE_{Zr})_M - (BE_{Zr})_A,$$

Supplementary Equation (3)

$$v_{\Delta BE} = (v_{BE})_A + (v_{BE})_M.$$

Supplementary Equation (4)

The BE_{Zr} metrics were calculated for the 13 studies that provided raw data for the performance of invaders and resident species.

To explore the potential mechanisms underlying the biodiversity effect on invasion resistance, we quantified transgressive resistance (analogous to transgressive overyielding in biodiversity–ecosystem functioning experiments[5]) using the equation:

$$\text{Transgressive resistance} = \ln(X_{\text{mono,low}}/X_{\text{maxmix,mean}}),$$

Supplementary Equation (5)

where $X_{\text{mono,low}}$ is the mean performance of invaders grown in the resident monoculture that has the lowest performance of invaders (i.e. has the highest invasion resistance), and $X_{\text{maxmix,mean}}$ is the mean performance of invaders in the most diverse mixture. Positive values of transgressive resistance indicate that the most diverse resident mixtures are more resistant to invasion than the most-resistant monoculture, indicating complementarity effects. Neutral values indicate that the most diverse resident mixtures are not more resistant than the most-resistant monoculture, indicating selection effects. Negative values are expected when there is no significant biodiversity effect on invasion resistance, as the invasion resistance of the mixture is the average of the invasion resistance of the individual resident species. The variance of transgressive resistance, v_{tran}, was calculated as:

$$v_{\text{tran}} = \frac{(S_{\text{mono,low}})^2}{n_{\text{mono,low}} \times (X_{\text{mono,low}})^2} + \frac{(S_{\text{maxmix,mean}})^2}{n_{\text{maxmix,mean}} \times (X_{\text{maxmix,mean}})^2}.$$

Supplementary Equation (6)

Transgressive resistance was calculated for the eight studies that provided raw data for each monoculture.

Similar to NBE, we used meta-regression models that included resident species richness, experimental duration and experimental unit size as covariates to test the effect of environmental manipulation (ambient vs. manipulated) on BE_{Zr} and transgressive resistance and to derive the mean effect size of ΔBE_{Zr}.

Supplementary Notes

Averaged across all studies, we found significantly positive invasion-resistance BE_{Zr} both under ambient and manipulated environmental conditions (Supplementary Figs. 1b, 17a). However, environmental change factors had negligible effects on invasion-resistance BE_{Zr}, except for drought, which decreased the strength of invasion-resistance BE_{Zr} ($Q_M = 6.04$, $p = 0.01$; mean $\Delta BE_{Zr} = -0.15$, 95% CI = [–0.27, –0.04]; Supplementary Fig. 17).

For the subset of studies for which we could calculate transgressive resistance of biodiversity to invasion, we found on average no significant transgressive resistance, nor an effect of environmental change factors on transgressive resistance (Supplementary Fig. 9). There was a positive effect size of transgressive resistance for the ambient environment of grazing studies, but this was based on only three observations (Supplementary Fig. 9).

For the subset of studies with data on resident productivity, we also found significantly positive effects of biodiversity on resident productivity under both ambient and manipulated environmental conditions (Supplementary Fig. 8). However, the strength of resident-productivity NBE was increased by drought ($Q_M = 15.98$, $p < 0.001$) but decreased by eutrophication ($Q_M = 11.99$, $p < 0.001$) (Supplementary Fig. 8a). The strength of resident-productivity BE_{Zr} was decreased by eutrophication ($Q_M = 10.09$, $p = 0.002$) and by all factors combined ($Q_M = 6.47$, $p = 0.01$) (Supplementary Fig. 8b). Both resident-productivity NBE ($Q_M = 11.73$, $p < 0.001$) and BE_{Zr} ($Q_M = 19.75$, $p < 0.001$) were decreased by two co-acting factors (Supplementary Fig. 8).

We found significantly positive relationships between invasion-resistance BE_{Zr} and resident-productivity BE_{Zr} under both ambient and manipulated environmental conditions (Supplementary Fig. 18a). Furthermore, we found that invasion-resistance BE_{Zr} increased with experimental duration under ambient but not under manipulated environmental conditions (Supplementary Fig. 18b).

We detected publication bias for invasion-resistance NBE (Supplementary Fig. 10a, b), but not for invasion-resistance BE_{Zr} (Supplementary Fig. 10c, d). Despite this, our sensitivity analysis showed that excluding outliers did not qualitatively affect our results (Supplementary Fig. 11), suggesting that the publication bias was unlikely to influence the robustness of our conclusions.

Supplementary Discussion

The effect of environmental change factors was weaker when we measured the biodiversity effect on invasion resistance as BE_{Zr} instead of NBE. Both measures have been widely used for quantifying biodiversity effects because they capture different attributes of the biodiversity–ecosystem functioning relationship[4]. NBE is more appropriate for cases where the ecosystem function saturates rapidly, while BE_{Zr} is more appropriate for cases where the ecosystem function increases linearly with species richness[4]. Therefore, we conjecture that environmental change factors affected invasion-resistance NBE by altering the saturation point, but had no effect on BE_{Zr} because invasion resistance changed to a similar degree across diversity treatments. Moreover, the non-significant effect of environmental change factors on invasion-resistance BE_{Zr} may also be attributed to the reduced statistical power as a result of the decreased sample size for this effect size metric.

Supplementary Table

Supplementary Table. 1 | Results of the Q_M tests for the effects of environmental manipulation, invader type and their interaction on the biodiversity effect (measured as NBE and BE_{Zr}) on invasion resistance.

Environmental change factors	Environmental manipulation		Invader type		Environmental manipulation * Invader type	
	Q_M	p	Q_M	p	Q_M	p
**NBE**						
All factors	0.22	0.64	43.18	<0.001	0.81	0.67
Warming	0.12	0.73	7.82	0.02	2.80	0.25
Elevated CO_2	2.69	0.10	9.63	0.002	2.56	0.11
Eutrophication	1.08	0.30	8.16	0.004	3.72	0.05
Pesticide	0.65	0.42	18.27	<0.001	2.21	0.33
Grazing	2.69	0.10	5.38	0.07	2.35	0.31
**BE_{Zr}**						
All factors	0.13	0.72	16.18	<0.001	0.29	0.86
Warming	0.07	0.79	1.61	0.45	1.17	0.56
Elevated CO_2	1.14	0.29	9.32	0.002	0.08	0.78
Eutrophication	4.97	0.03	10.59	0.001	2.45	0.12
Pesticide	0.13	0.72	4.08	0.13	0.84	0.66
Grazing	0.71	0.40	12.14	0.002	2.26	0.32

Note: NBE, the net biodiversity effect measured by the natural log of the response ratio; BE_{Zr}, the biodiversity effect measured by Fisher's z-transformed correlation coefficient.

Supplementary Figures

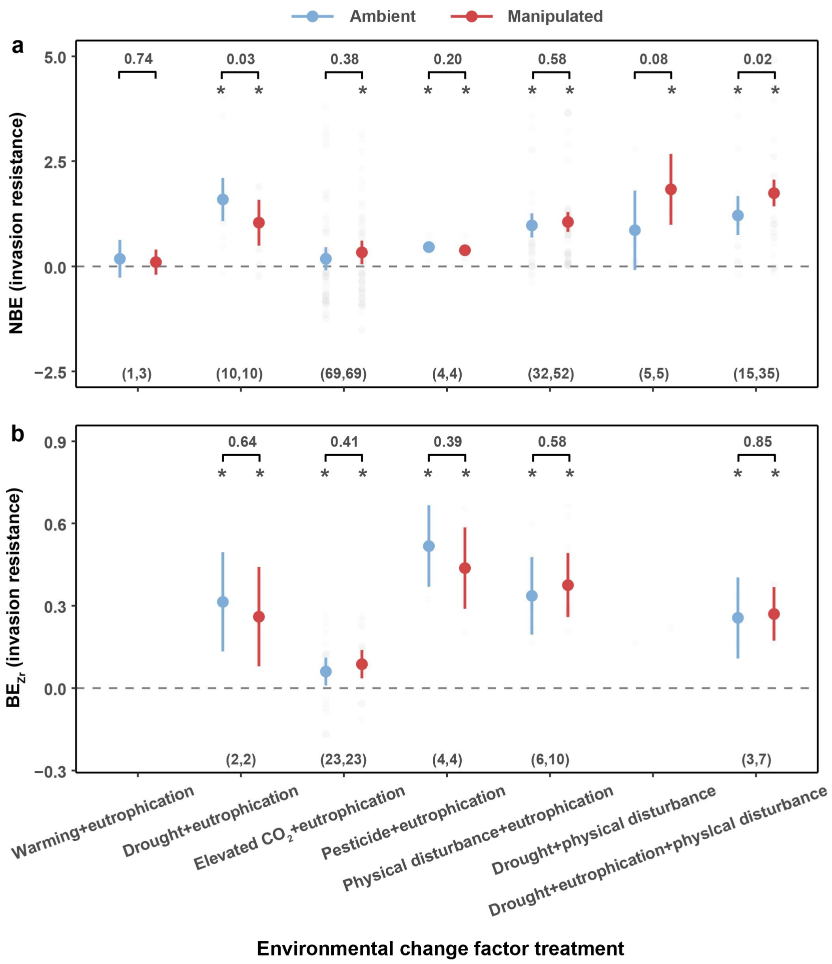

Supplementary Fig. 1 | The biodiversity effect on invasion resistance, measured as NBE (a) and BE_{Zr} (b), in ambient and manipulated environments exposed to multiple environmental change factors. NBE is the net biodiversity effect measured by the natural log of the response ratio, BE_{Zr} is the biodiversity effect measured by Fisher's z-transformed correlation coefficient. Positive values of NBE or BE_{Zr} indicate higher invasion resistance of resident mixtures in comparison with that of resident monocultures, whereas negative values indicate the opposite. The numbers above the brackets are the *p*-values of the Q_M tests for the effect of environmental manipulation (ambient vs. manipulated) on NBE or BE_{Zr}. The numbers in brackets show the number of effect sizes. Points with error bars are the estimated means and corrected 95% confidence intervals. Confidence intervals not overlapping with the dashed line (i.e. 0) indicate statistical significance, as indicated by asterisks.

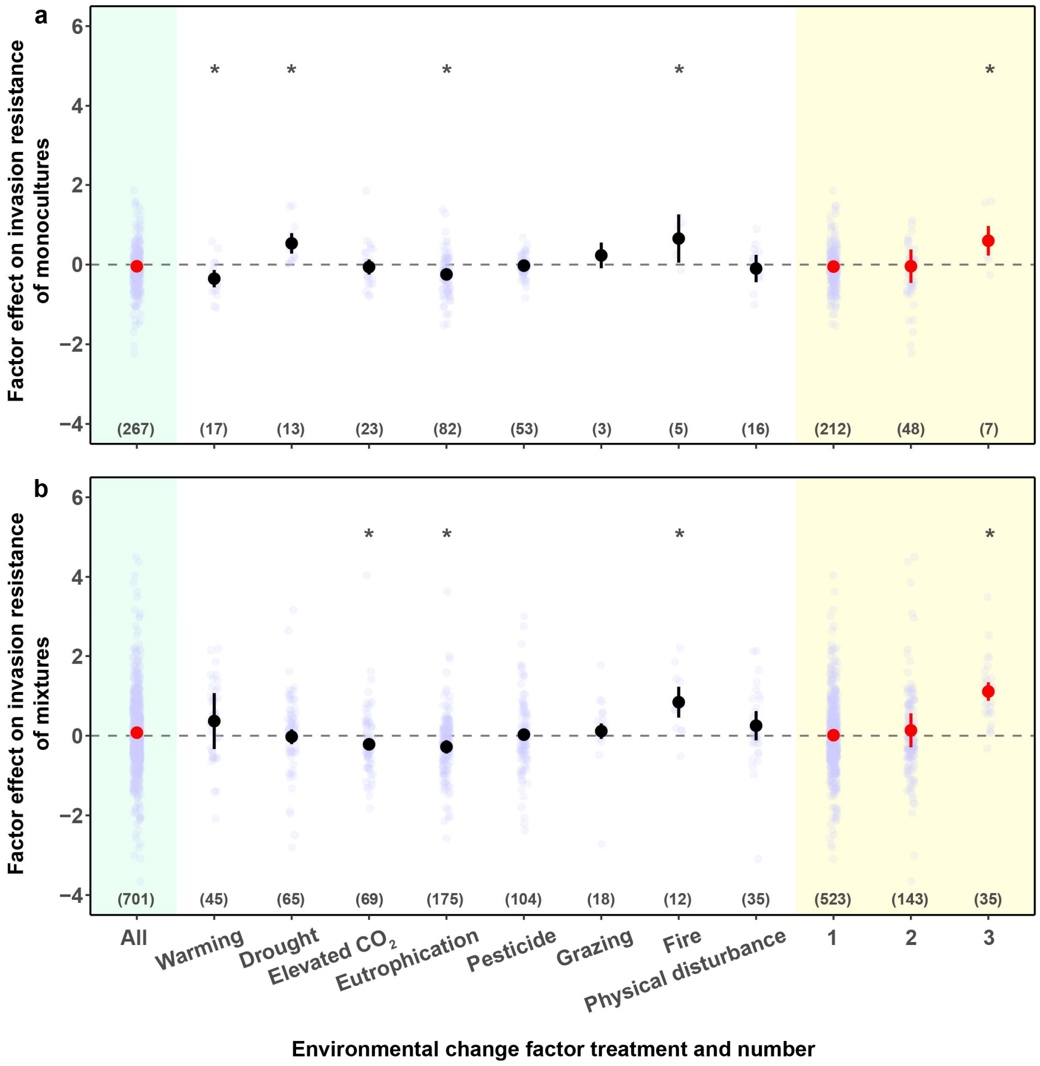

Supplementary Fig. 2 | Effects of environmental change factors on invasion resistance of resident monocultures (a) and mixtures (b). Positive values of the factor effect on invasion resistance indicate that environmental change factors increase invasion resistance, whereas negative values indicate the opposite. The numbers in brackets show the number of effect sizes. Points with error bars are the estimated means and corrected 95% confidence intervals. Confidence intervals not overlapping with the dashed line (i.e. 0) indicate statistical significance, as indicated by asterisks. Green shading indicates the analysis on all environmental change factors and yellow shading indicates the analysis on different numbers of factors.

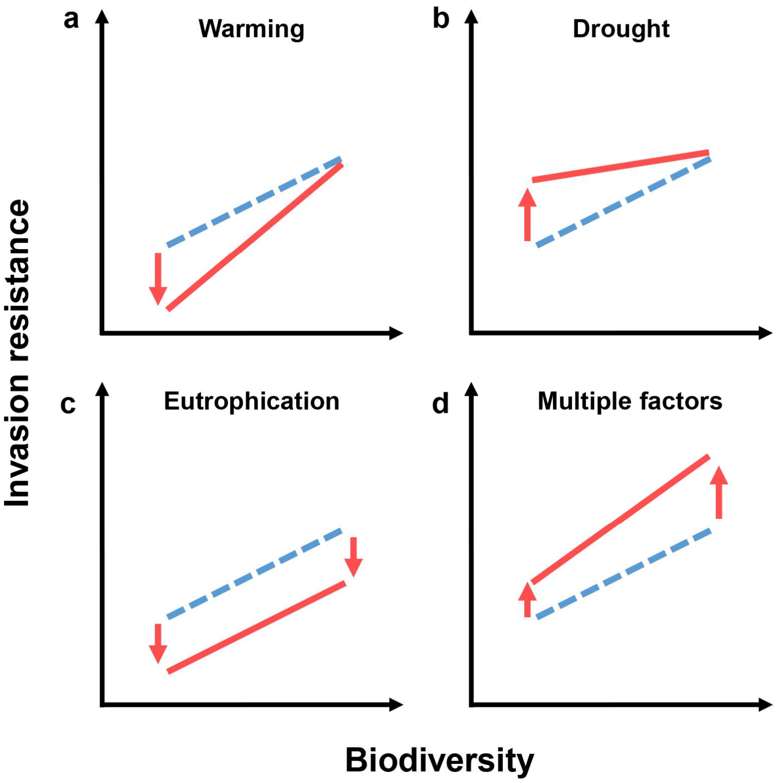

Supplementary Fig. 3 | A summary of the net biodiversity effect on invasion resistance in the face of warming (a), drought (b), eutrophication (c) and multiple co-acting environmental change factors (d). Blue dashed lines represent the relationship between biodiversity and invasion resistance under ambient conditions. Red solid lines represent the relationship between biodiversity and invasion resistance under manipulated environmental conditions.

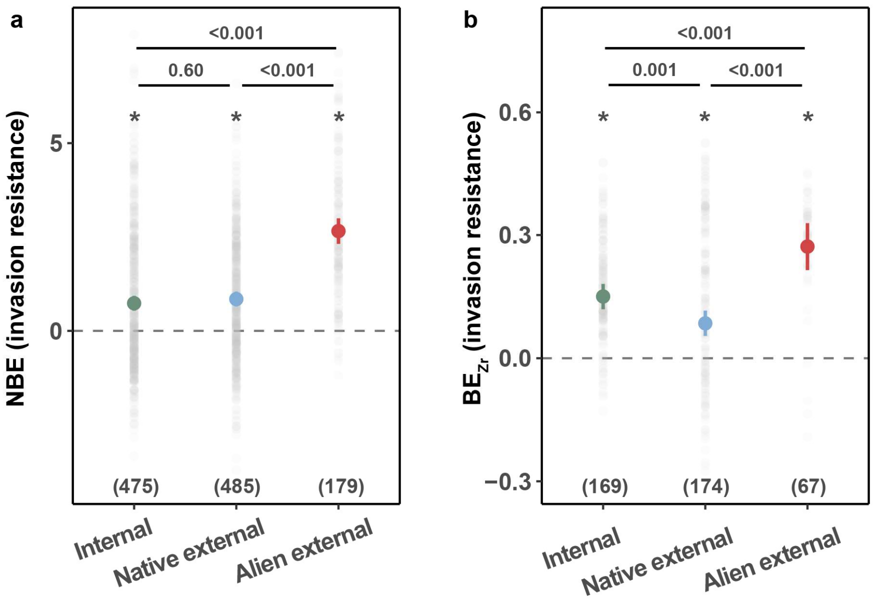

Supplementary Fig. 4 | The biodiversity effect on invasion resistance, measured as NBE (a) and BE_{Zr} (b), among invader types. NBE is the net biodiversity effect measured by the natural log of the response ratio, BE_{Zr} is the biodiversity effect measured by Fisher's z-transformed correlation coefficient. Positive values of NBE or BE_{Zr} indicate higher invasion resistance of resident mixtures in comparison with that of resident monocultures, whereas negative values indicate the opposite. The numbers above the lines are the *p*-values. The numbers in brackets show the number of effect sizes. Points with error bars are the estimated means and corrected 95% confidence intervals. Confidence intervals not overlapping with the dashed line (i.e. 0) indicate statistical significance, as indicated by asterisks.

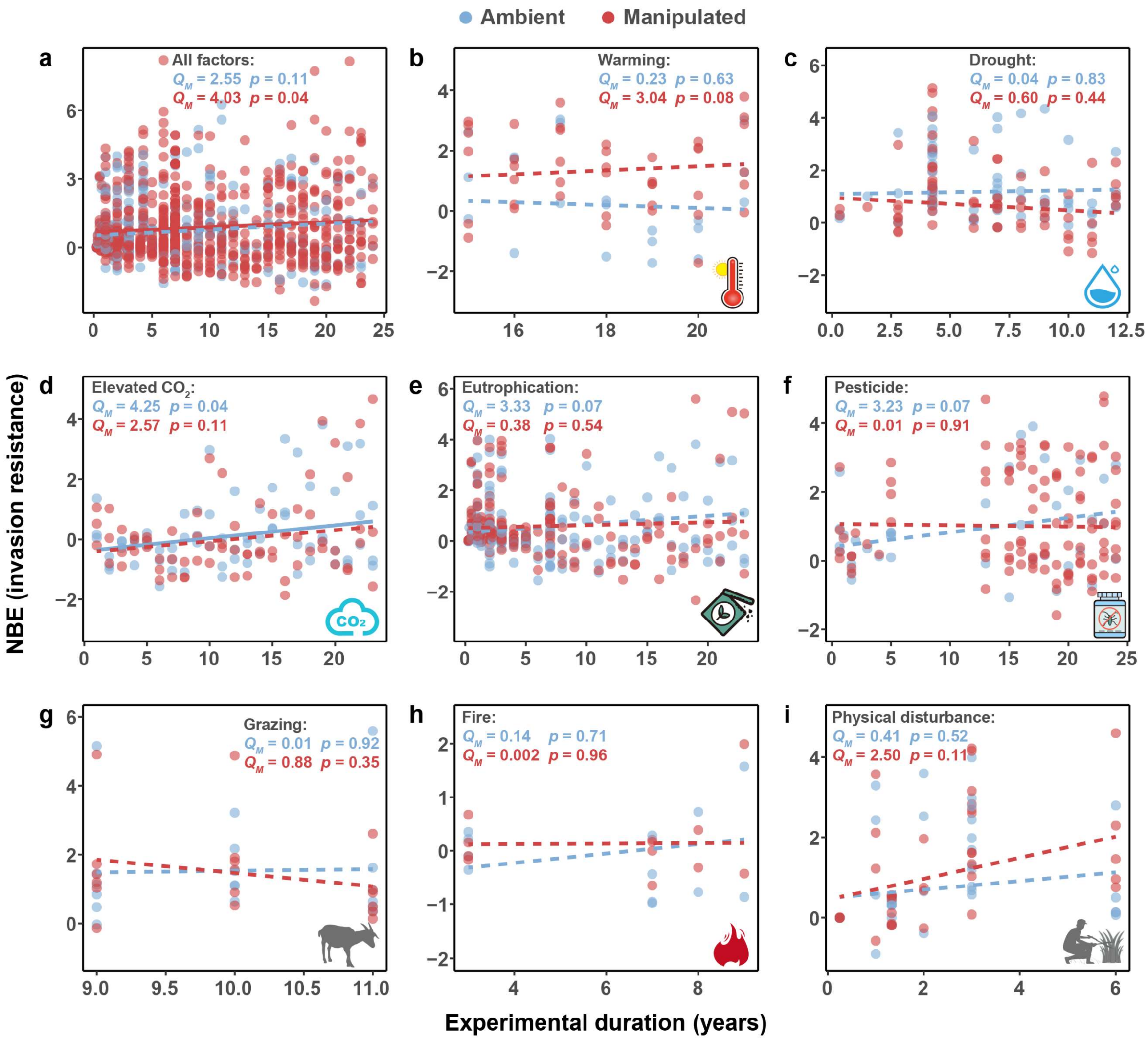

Supplementary Fig. 5 | Relationships between the net biodiversity effect (NBE) on invasion resistance and experimental duration. Relationships were tested using the Q_M tests for datasets of all environmental change factors (**a**), warming (**b**), drought (**c**), elevated CO_2 (**d**), eutrophication (**e**), pesticide (**f**), grazing (**g**), fire (**h**) and physical disturbance (**i**). Positive values of NBE indicate higher invasion resistance of resident mixtures in comparison with that of resident monocultures, whereas negative values indicate the opposite. Blue indicates the ambient condition and red indicates the manipulated environmental condition. Symbols of environmental change factors are created by Yue Chen.

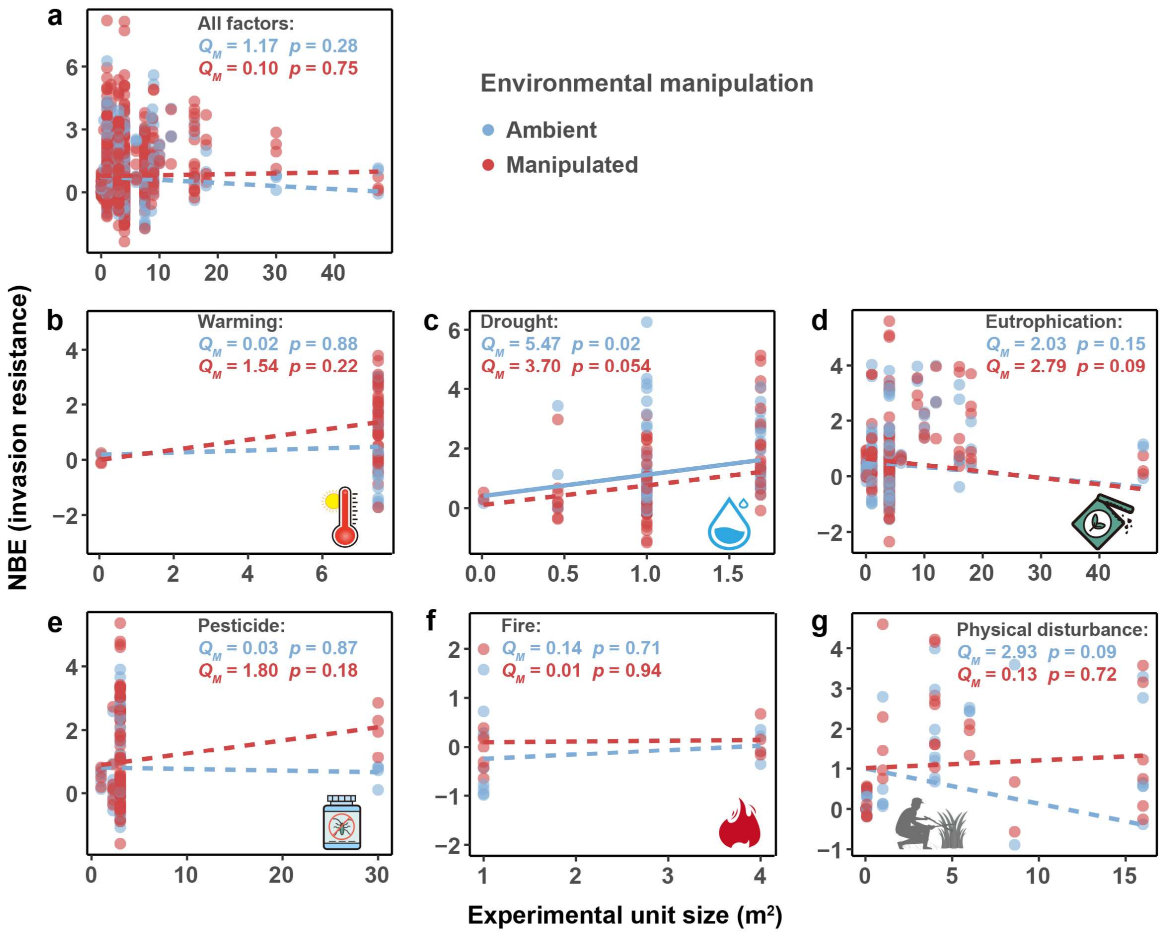

Supplementary Fig. 6 | Relationships between the net biodiversity effect (NBE) on invasion resistance and experimental unit size. Relationships were tested using the Q_M tests for datasets of all environmental change factors (**a**), warming (**b**), drought (**c**), eutrophication (**d**), pesticide (**e**), fire (**f**) and physical disturbance (**g**). Positive values of NBE indicate higher invasion resistance of resident mixtures in comparison with that of resident monocultures, whereas negative values indicate the opposite. Blue indicates the ambient condition and red indicates the manipulated environmental condition. Symbols of environmental change factors are created by Yue Chen.

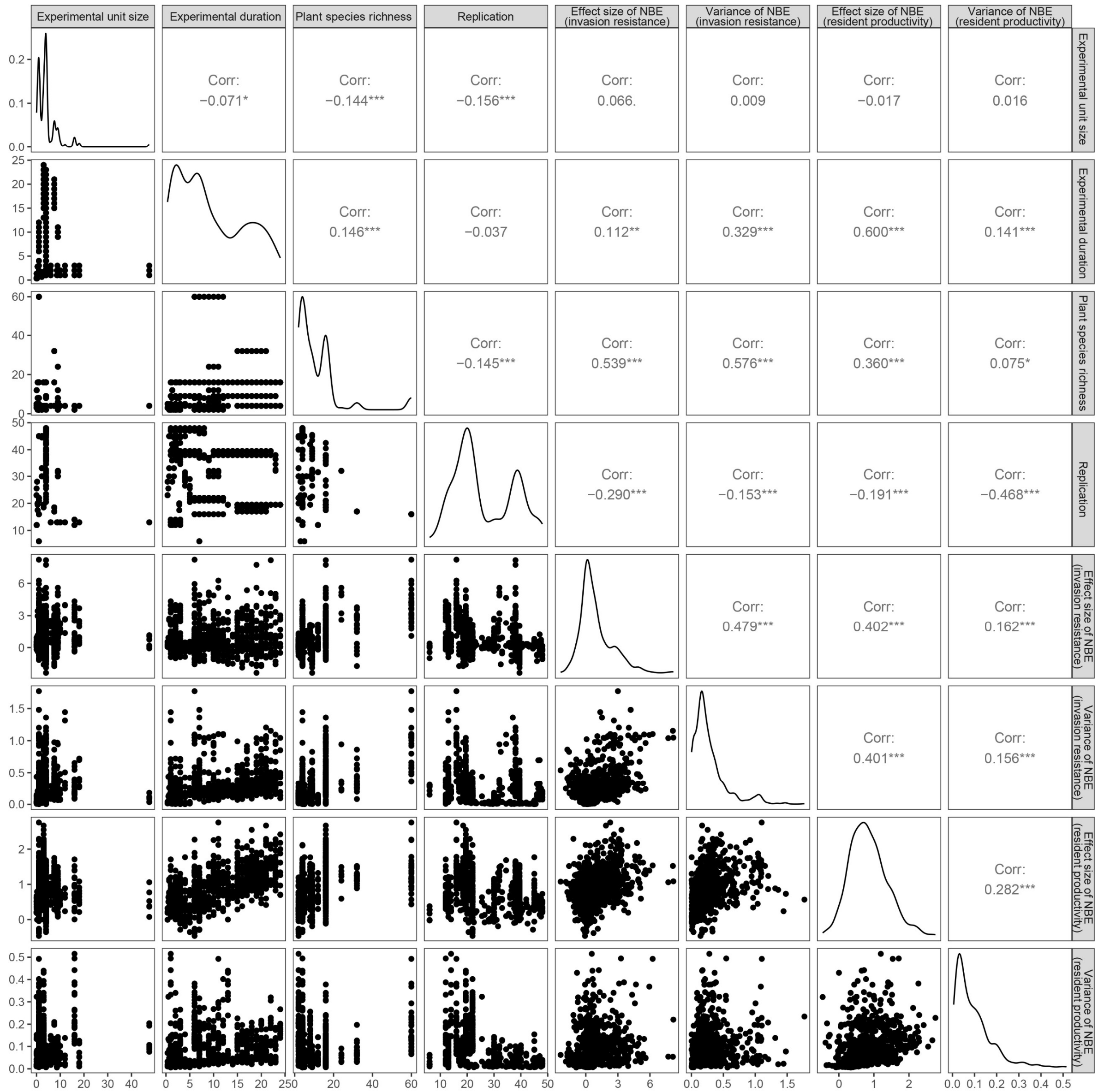

Supplementary Fig. 7 | Correlations among experimental unit size, experimental duration, resident plant species richness, replication, effect size and variance of net biodiversity effect (NBE). *** $p < 0.001$, ** $p < 0.01$, * $p < 0.05$.

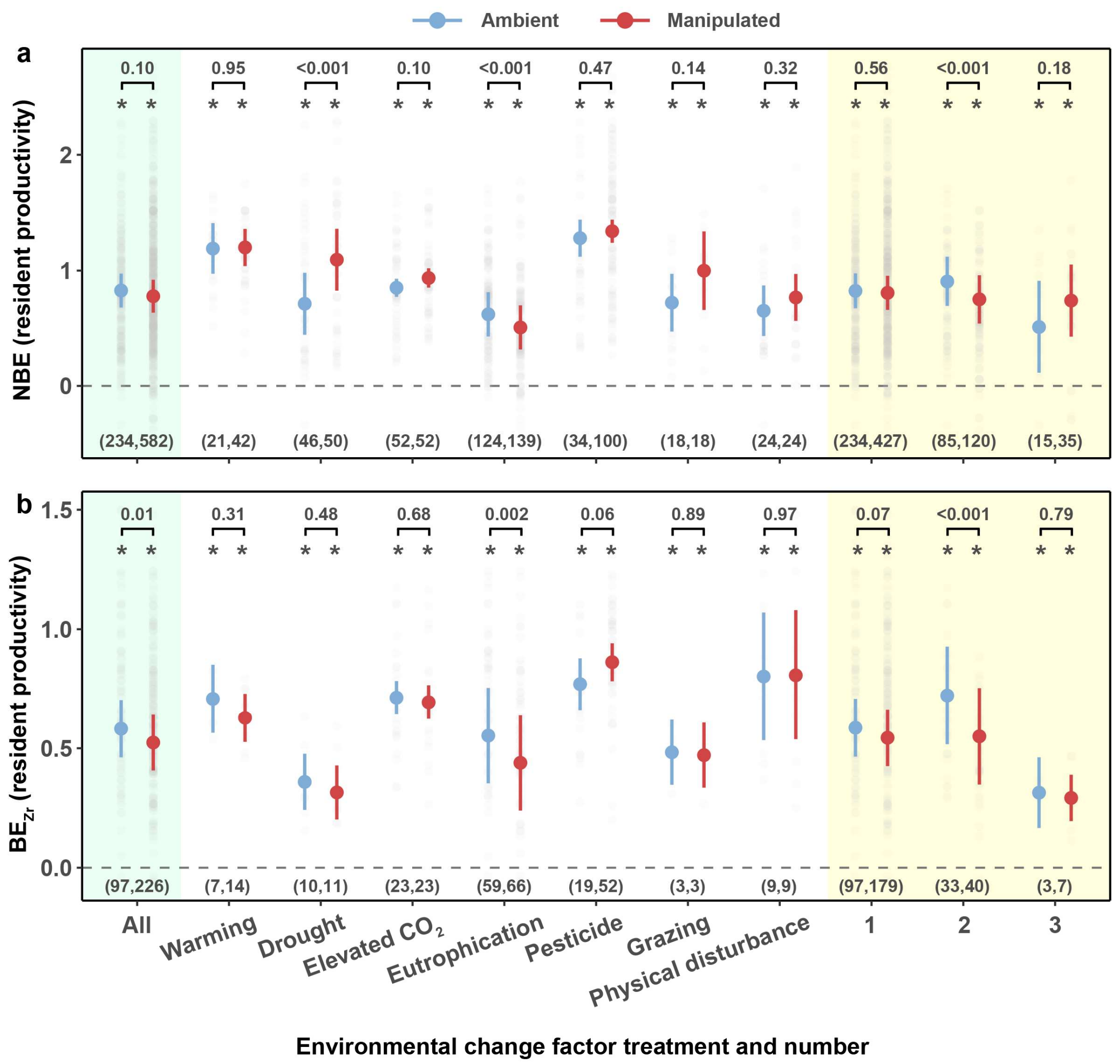

Supplementary Fig. 8 | The biodiversity effect on resident productivity, measured as NBE (a) and BE$_{Zr}$ (b), under ambient and manipulated environmental conditions. NBE is the net biodiversity effect measured by the natural log of the response ratio, BE$_{Zr}$ is the biodiversity effect measured by Fisher's z-transformed correlation coefficient. Positive values of NBE or BE$_{Zr}$ indicate higher productivity of resident mixtures in comparison to resident monocultures, whereas negative values indicate the opposite. The numbers above the brackets are the p-values of the Q_M tests for the effect of environmental manipulation (ambient vs. manipulated) on NBE or BE$_{Zr}$. The numbers in brackets show the number of effect sizes. Points with error bars are the estimated means and corrected 95% confidence intervals. Confidence intervals not overlapping with the dashed line (i.e. 0) indicate statistical significance, as indicated by asterisks. Green shading indicates the analysis on all environmental change factors and yellow shading indicates the analysis on different numbers of factors.

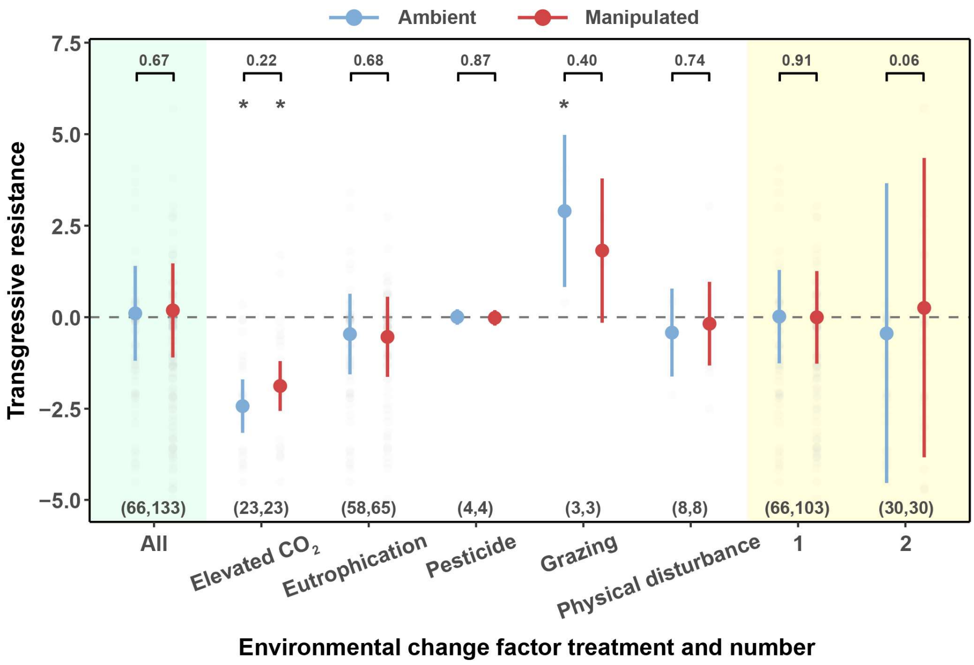

Supplementary Fig. 9 | Transgressive resistance of biodiversity under ambient and manipulated environmental conditions. Positive values of transgressive resistance indicate that the most diverse resident mixtures are more resistant to invasion than the most-resistant monoculture, indicating complementarity effects. Neutral values indicate that the most diverse resident mixtures are not more resistant than the most-resistant monoculture, indicating selection effects. Negative values are expected when there is no significant biodiversity effect on invasion resistance, as the invasion resistance of the mixture is the average of the invasion resistance of the individual resident species. The numbers above the brackets are the *p*-values of the Q_M tests for the effect of environmental manipulation (ambient vs. manipulated) on transgressive resistance. The numbers in brackets show the number of effect sizes. Points with error bars are the estimated means and corrected 95% confidence intervals. Confidence intervals not overlapping with the dashed line (i.e. 0) indicate statistical significance, as indicated by asterisks. Green shading indicates the analysis on all environmental change factors and yellow shading indicates the analysis on different numbers of factors.

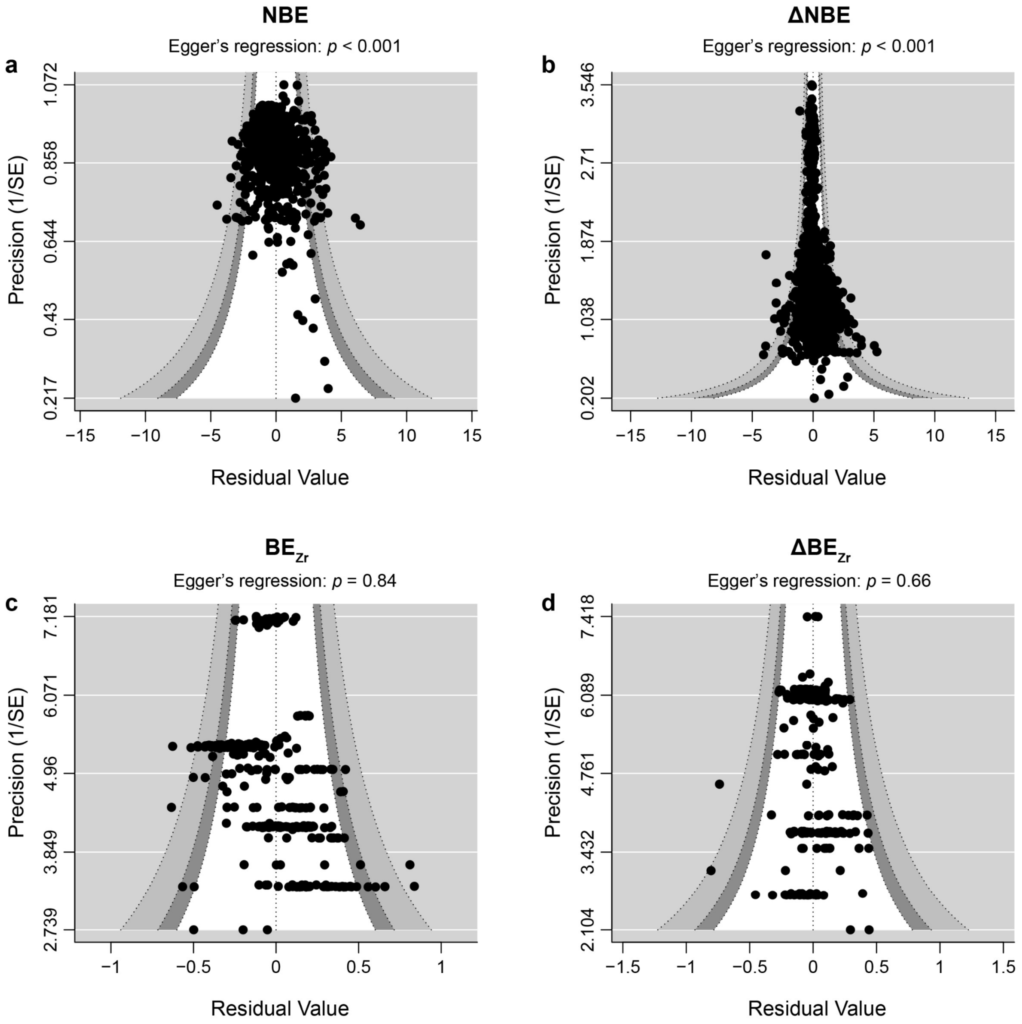

Supplementary Fig. 10 | Funnel plots for model residuals and Egger's test on the biodiversity effect on invasion resistance, measured as NBE (a, b) and BE_{Zr} (c, d). NBE is the net biodiversity effect measured by the natural log of the response ratio, BE_{Zr} is the biodiversity effect measured by Fisher's z-transformed correlation coefficient. ΔNBE or ΔBE_{Zr} is the difference in NBE or BE_{Zr} between manipulated and ambient conditions.

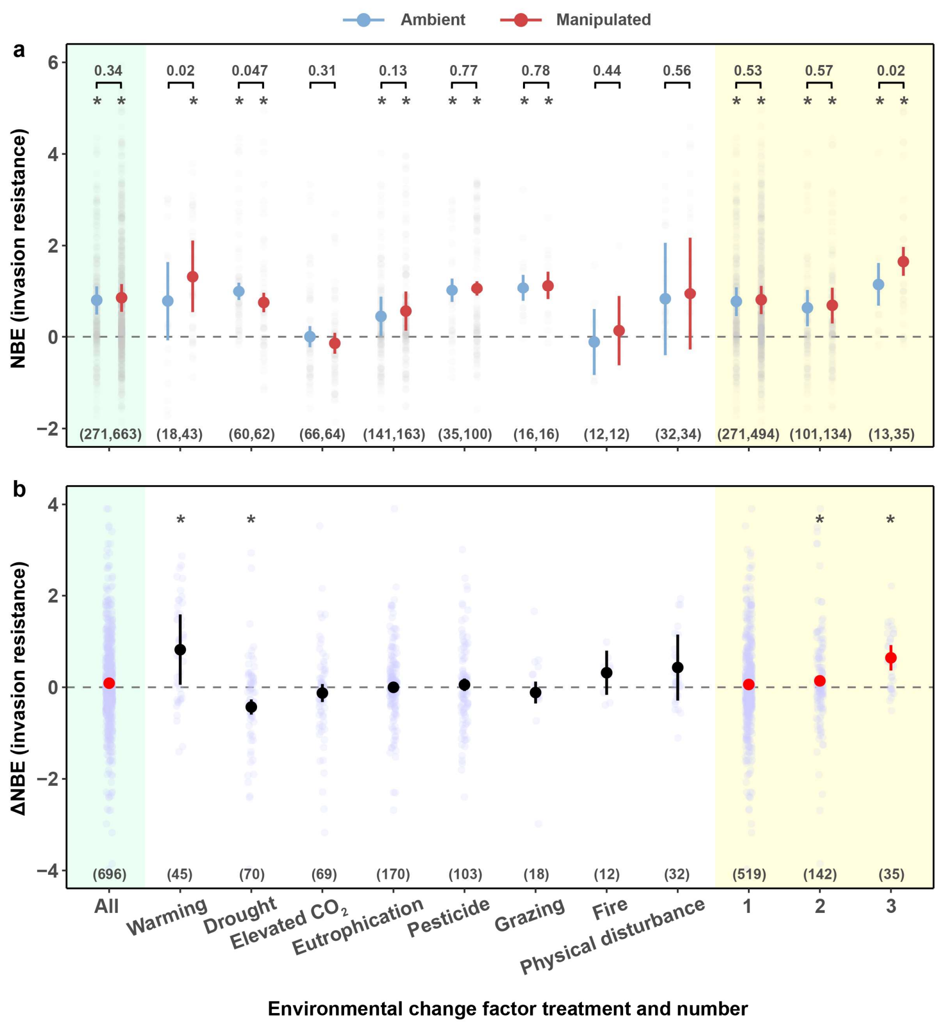

Supplementary Fig. 11 | The net biodiversity effect (NBE) on invasion resistance (a) and the difference in NBE between ambient and manipulated environmental conditions (ΔNBE) (b) after excluding outliers. Positive values of NBE indicate higher invasion resistance of resident mixtures in comparison with that of resident monocultures, whereas negative values indicate the opposite. Positive values of ΔNBE indicate stronger biodiversity effects under manipulated environmental conditions in comparison with ambient conditions, and vice versa. In panel **a**, the numbers above the brackets are the p-values of the Q_M tests for the effect of environmental manipulation (ambient vs. manipulated) on NBE. The numbers in brackets show the number of effect sizes. Points with error bars are the estimated means with corrected 95% confidence intervals. Confidence intervals not overlapping with the dashed line (i.e. 0) indicate statistical significance, as indicated by asterisks. Green shading indicates the analysis on all environmental change factors and yellow shading indicates the analysis on different numbers of factors.

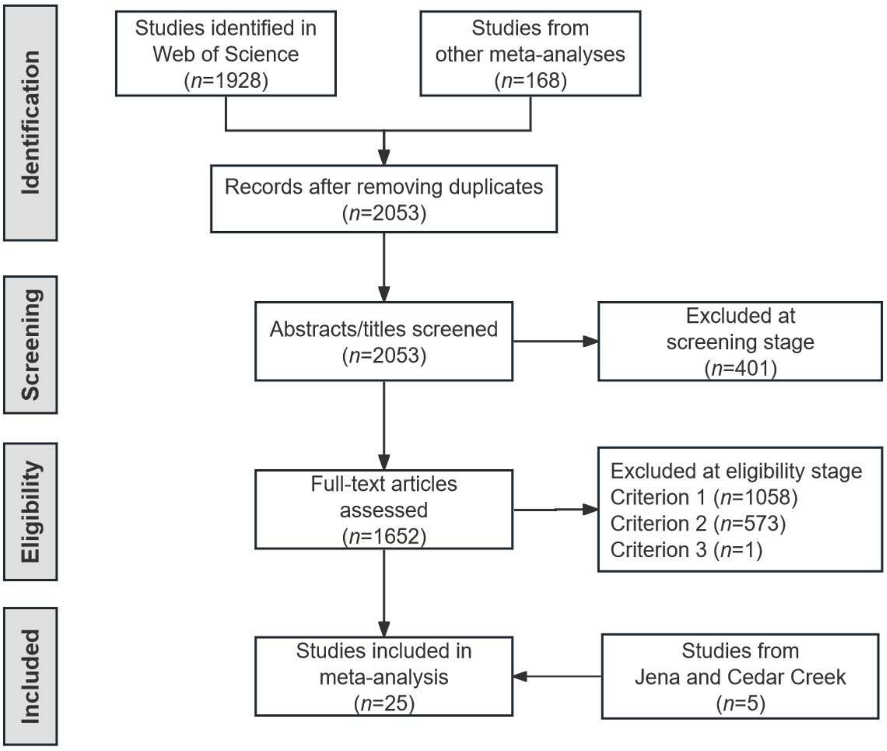

Supplementary Fig. 12 | PRISMA flow diagram showing the processes of identification, screening, eligibility, and inclusion of studies. Eligibility criteria include: (1) the study must have manipulated the number of species in the resident community directly (i.e. observational studies were excluded); (2) the study must have compared mixtures with monocultures under both ambient and manipulated environmental conditions; (3) the study must provide the mean, statistical variation (standard deviation, standard error or 95% confidence intervals), and sample sizes for the performance of invaders (including both alien and native species) in different treatments.

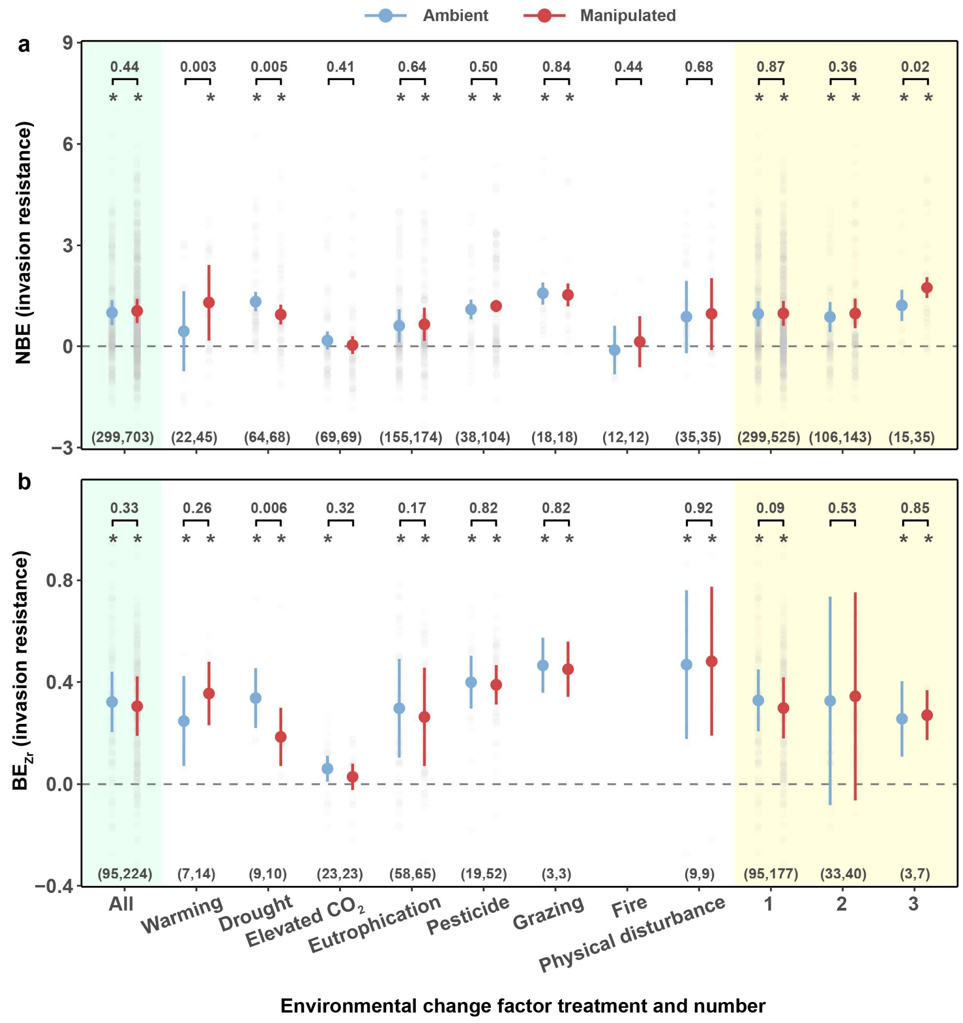

Supplementary Fig. 13 | The biodiversity effect on invasion resistance, measured as NBE (a) and BE_{Zr} (b), under ambient and manipulated environmental conditions after excluding greenhouse studies. NBE is the net biodiversity effect measured by the natural log of the response ratio, BE_{Zr} is the biodiversity effect measured by Fisher's z-transformed correlation coefficient. Positive values of NBE or BE_{Zr} indicate higher invasion resistance of resident mixtures in comparison to resident monocultures, whereas negative values indicate the opposite. The numbers above the brackets are the *p*-values of the Q_M tests for the effect of environmental manipulation (ambient vs. manipulated) on NBE or BE_{Zr}. The numbers in brackets show the number of effect sizes. Points with error bars are the estimated means and corrected 95% confidence intervals. Confidence intervals not overlapping with the dashed line (i.e. 0) indicate statistical significance, as indicated by asterisks. Green shading indicates the analysis on all environmental change factors and yellow shading indicates the analysis on different numbers of factors.

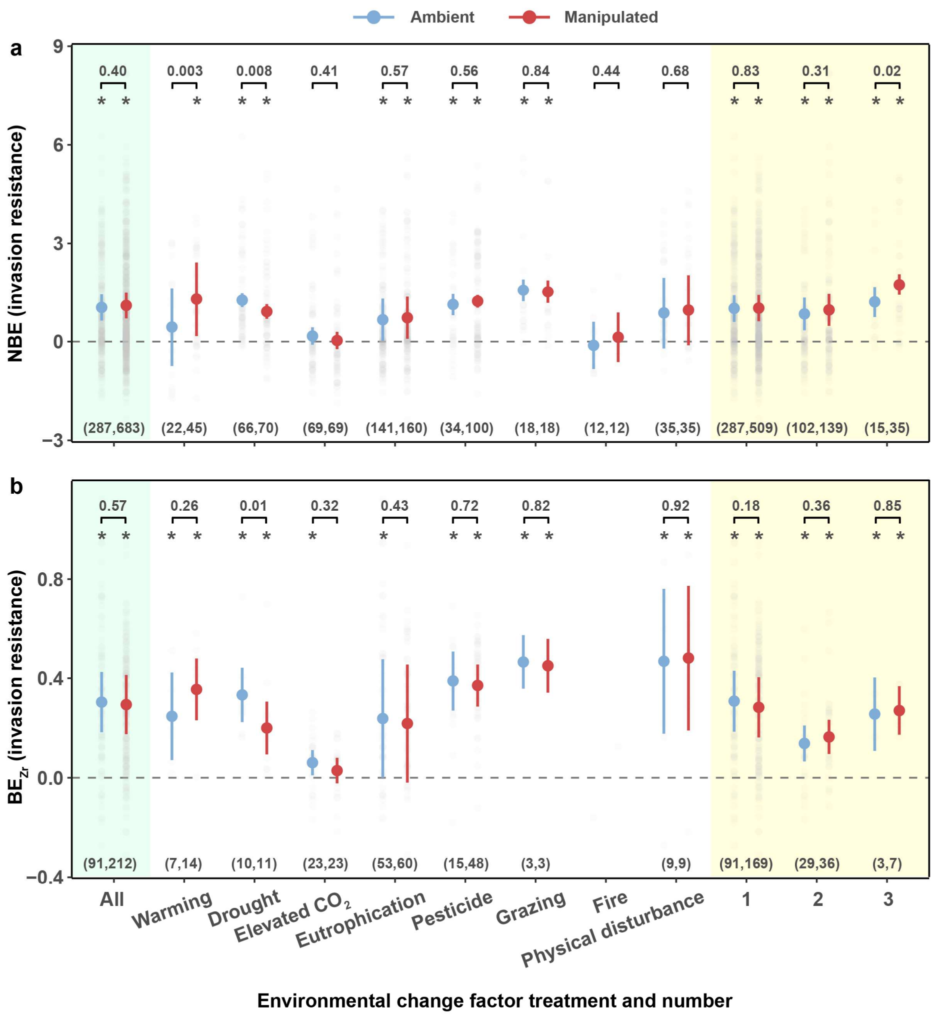

Supplementary Fig. 14 | The biodiversity effect on invasion resistance, measured as NBE (a) and BE_{Zr} (b), under ambient and manipulated environmental conditions after excluding cover data. NBE is the net biodiversity effect measured by the natural log of the response ratio, BE_{Zr} is the biodiversity effect measured by Fisher's z-transformed correlation coefficient. Positive values of NBE or BE_{Zr} indicate higher invasion resistance of resident mixtures in comparison to resident monocultures, whereas negative values indicate the opposite. The numbers above the brackets are the *p*-values of the Q_M tests for the effect of environmental manipulation (ambient vs. manipulated) on NBE or BE_{Zr}. The numbers in brackets show the number of effect sizes. Points with error bars are the estimated means and corrected 95% confidence intervals. Confidence intervals not overlapping with the dashed line (i.e. 0) indicate statistical significance, as indicated by asterisks. Green shading indicates the analysis on all environmental change factors and yellow shading indicates the analysis on different numbers of factors.

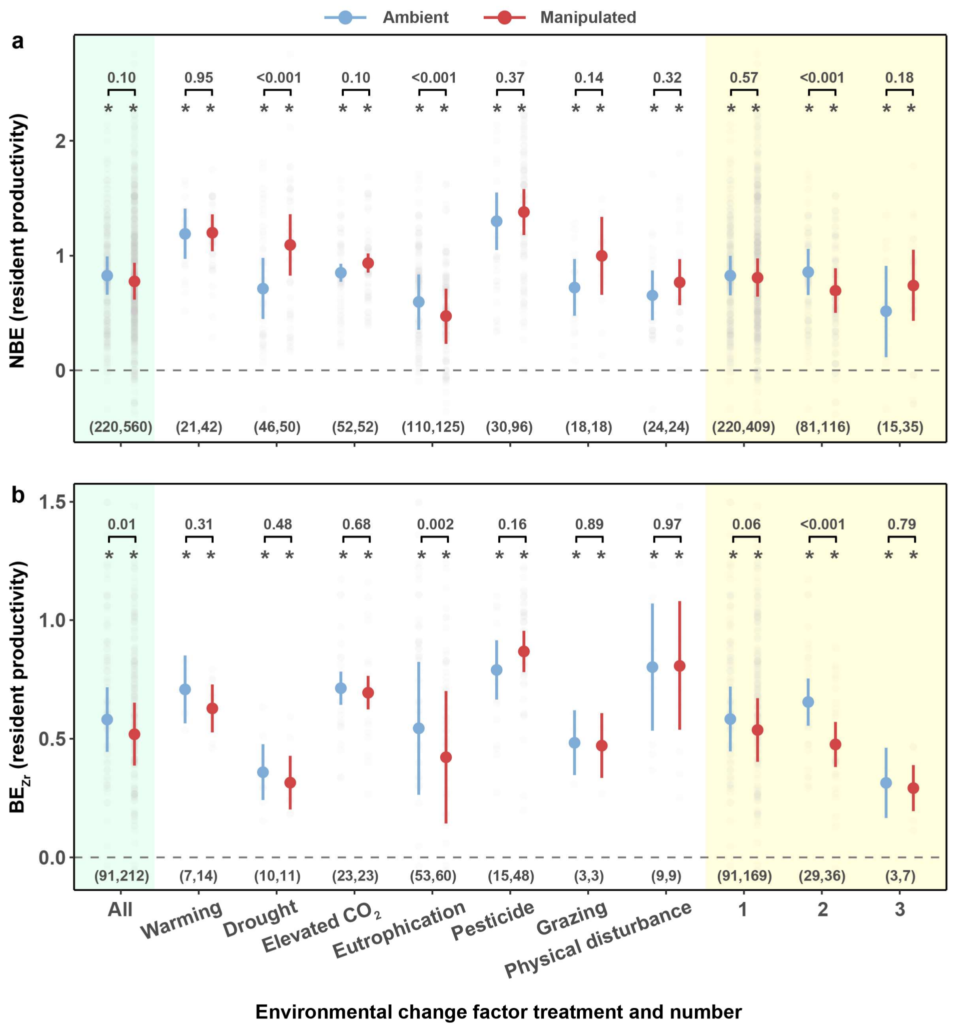

Supplementary Fig. 15 | The biodiversity effect on resident productivity, measured as NBE (a) and BE_{Zr} (b), under ambient and manipulated environmental conditions after excluding cover data. NBE is the net biodiversity effect measured by the natural log of the response ratio, BE_{Zr} is the biodiversity effect measured by Fisher's z-transformed correlation coefficient. Positive values of NBE or BE_{Zr} indicate higher productivity of resident mixtures in comparison to resident monocultures, whereas negative values indicate the opposite. The numbers above the brackets are the *p*-values of the Q_M tests for the effect of environmental manipulation (ambient vs. manipulated) on NBE or BE_{Zr}. The numbers in brackets show the number of effect sizes. Points with error bars are the estimated means and corrected 95% confidence intervals. Confidence intervals not overlapping with the dashed line (i.e. 0) indicate statistical significance, as indicated by asterisks. Green shading indicates the analysis on all environmental change factors and yellow shading indicates the analysis on different numbers of factors.

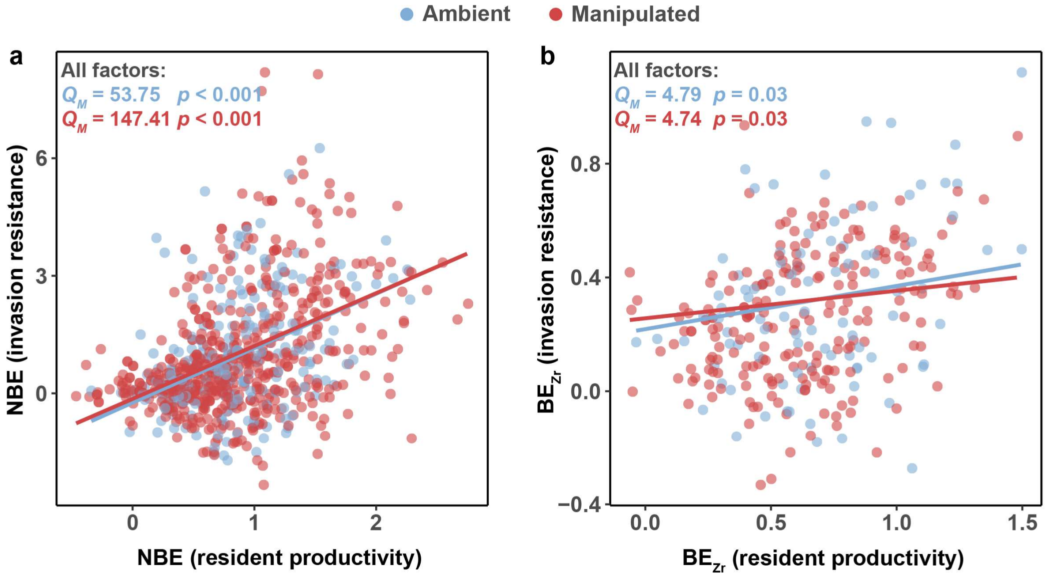

Supplementary Fig. 16 | Relationships between the biodiversity effect on invasion resistance, measured as NBE (a) and BE_{Zr} (b), and the effect on resident productivity after excluding cover data. NBE is the net biodiversity effect measured by the natural log of the response ratio, BE_{Zr} is the biodiversity effect measured by Fisher's z-transformed correlation coefficient. Positive values of NBE or BE_{Zr} indicate higher invasion resistance or productivity of resident mixtures in comparison with that of resident monocultures, whereas negative values indicate the opposite. Relationships were tested using the Q_M tests. Blue indicates the ambient condition and red indicates the manipulated environmental condition.

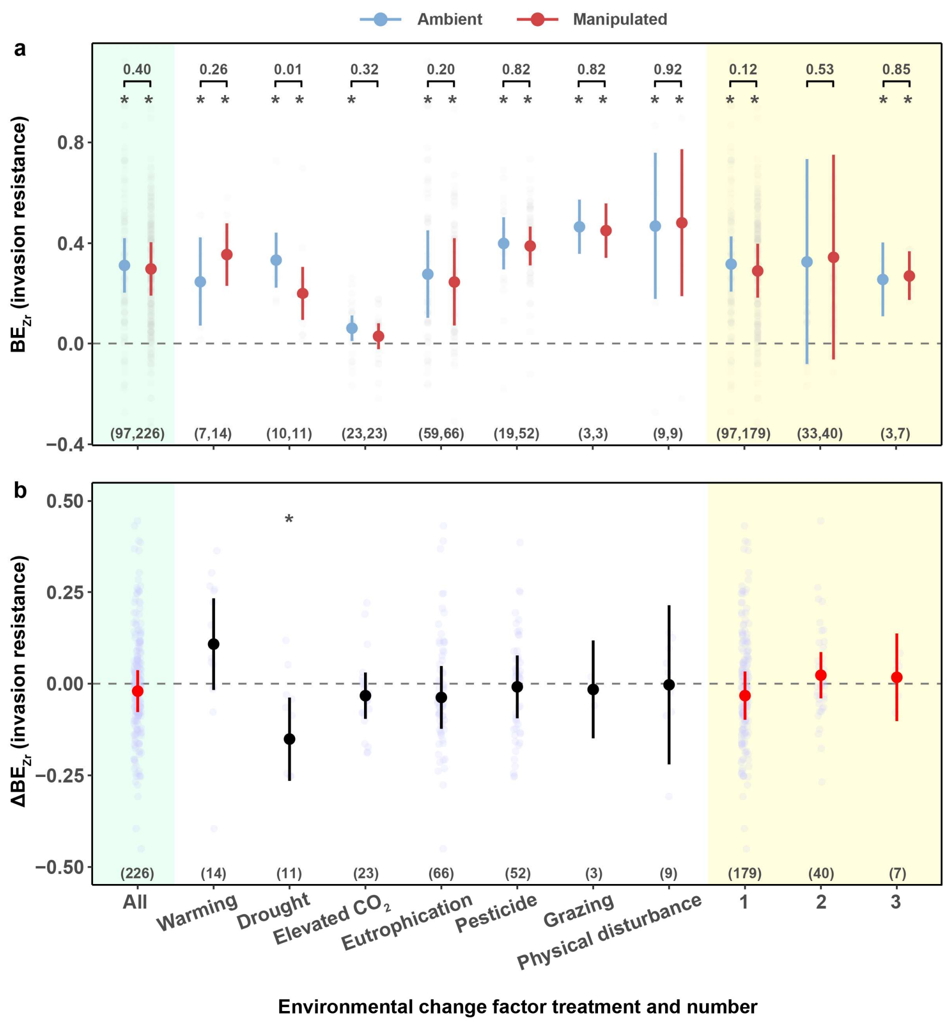

Supplementary Fig. 17 | The biodiversity effect (measured as BE_{Zr}) on invasion resistance (a) and the difference in BE_{Zr} between ambient and manipulated environmental conditions (ΔBE_{Zr}) (b). Positive values of BE_{Zr} indicate higher invasion resistance of resident mixtures in comparison with that of resident monocultures, whereas negative values indicate the opposite. Positive values of ΔBE_{Zr} indicate stronger biodiversity effects under manipulated environmental conditions in comparison to ambient conditions, and vice versa. In panel **a**, the numbers above the brackets are the *p*-values of the Q_M tests for the effect of environmental manipulation (ambient vs. manipulated) on BE_{Zr}. The numbers in brackets show the number of effect sizes. Points with error bars are the estimated means and corrected 95% confidence intervals. Confidence intervals not overlapping with the dashed line (i.e. 0) indicate statistical significance, as indicated by asterisks. Green shading indicates the analysis on all environmental change factors and yellow shading indicates the analysis on different numbers of factors.

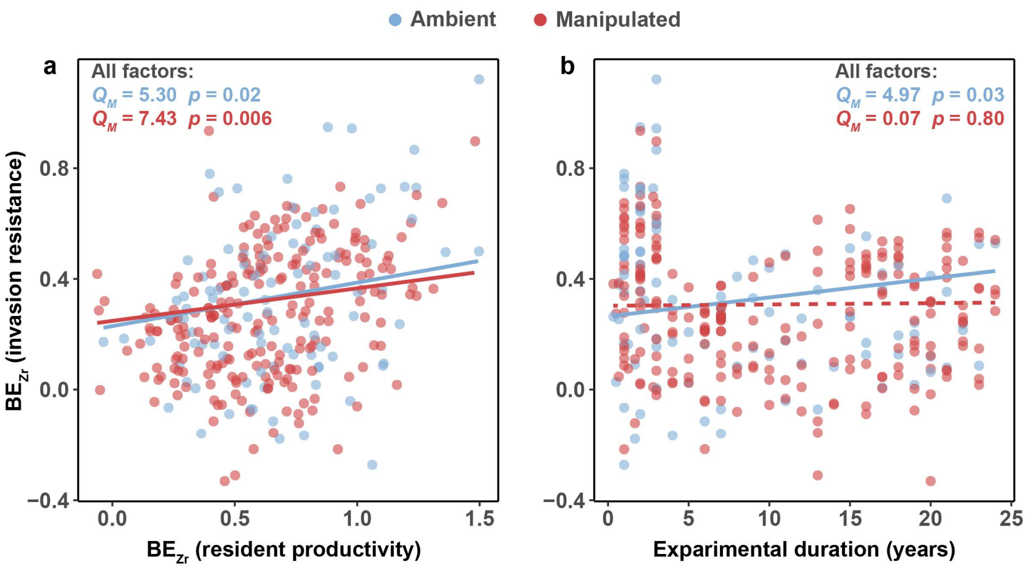

Supplementary Fig. 18 | Relationships between the biodiversity effect (measured as BE_{Zr}) on invasion resistance and the effect on resident productivity (a) and experimental duration (b). Positive values of BE_{Zr} indicate higher invasion resistance or productivity of resident mixtures in comparison with that of resident monocultures, whereas negative values indicate the opposite. Relationships were tested using the Q_M tests. Blue indicates the ambient condition and red indicates the manipulated environmental condition.

Supplementary References

1. Peng, S., Kinlock, N. L., Gurevitch, G. & Peng, S. Correlation of native and exotic species richness: a global meta-analysis finds no invasion paradox across scales. *Ecology* **100**, e02552 (2019).
2. Smith, N. S. & Côté, I. M. Multiple drivers of contrasting diversity–invasibility relationships at fine spatial grains. *Ecology* **100**, e02573 (2019).
3. Borenstein, M. et al. *Introduction to Meta-analysis* (John Wiley & Sons, Hoboken, 2009).
4. Hong, P. et al. Biodiversity promotes ecosystem functioning despite environmental change. *Ecol. Lett.* **25**, 555–569 (2022).
5. Hector, A., Bazeley-White, E., Loreau, M., Otway, S. & Schmid, B. Overyielding in grassland communities: testing the sampling effect hypothesis with replicated biodiversity experiments. *Ecol. Lett.* **5**, 502–511 (2002).
