# Supplementary material for: Biodiversity increases resistance of grasslands against plant invasions under multiple environmental changes
Source: Nat Commun. 2024 May 27;15:4506. doi: 10.1038/s41467-024-48876-z (PMC11130343; doi:10.1038/s41467-024-48876-z)
Supplement: Supplementary file 3 — Description of Additional Supplementary Files [file 41467_2024_48876_MOESM3_ESM.pdf]

Description of Additional Supplementary Files

File Name: Supplementary Data 1
Description: This dataset includes all studies used in this meta-analysis.
